# LncRNA LINC00588 Suppresses the Progression of Osteosarcoma by Acting as a ceRNA for miRNA-1972

**DOI:** 10.3389/fphar.2020.00255

**Published:** 2020-03-24

**Authors:** Fu-Chao Zhou, Yue-Hui Zhang, Hai-Tao Liu, Jia Song, Jiang Shao

**Affiliations:** Spine Center, Xin Hua Hospital Affiliated to Shanghai Jiao Tong University School of Medicine, Shanghai, China

**Keywords:** lncRNA LINC00588, miRNA-1972, TP53, osteosarcoma, ceRNA

## Abstract

Long non-coding RNAs (lncRNAs) are being found to play an increasingly important role in the development of tumors. However, their biological functions and the underlying mechanisms remain unclear. Using information from GEO Datasets, we found that the lncRNA LINC00588 was downregulated in osteosarcoma (OS) in bone but was upregulated in the metastatic tumor present in the lung. We assessed the function of LINC00588 using both overexpression and knock-out studies. We performed colony formation assay, CCK-8 assay, flow cytometry, wound healing assay, transwell assay, and RT-qPCR assay and used a xenograft model to investigate the influence of LINC00588 on cell proliferation, viability, cell apoptosis and cycle, migration, invasion, endothelial cell function, EMT (epithelial to mesenchymal transition), and tumor growth, respectively. Overexpression of LINC00588 appeared to inhibit cell proliferation, viability, migration, invasion, endothelial cell function, EMT, and tumor growth but not apoptosis, while we got the opposite result when we knocked down LINC00588. Next, we predicted that LINC00588 bound to miRNA-1972 and significantly downregulated its expression, which we then verified through a luciferase reporter assay. Subsequently, we knocked down miR1972 and performed CCK-8 and transwell assays to demonstrate that downregulation of miRNA-1972 could substantially inhibit the viability and invasion of osteosarcoma cells. The expression of TP53 was downregulated at the protein level but not at the mRNA level after the overexpression of miRNA-1972. Taken together, our findings indicate that LINC00588 plays a role in OS development by downregulating the expression of miRNA-1972, which can, in turn, inhibit the expression of TP53. Hence, we believe that the LINC00588/miRNA-1072/TP53 axis could potentially serve as a therapeutic target or diagnostic biomarker for osteosarcoma.

## Introduction

Osteosarcoma (OS) is the most common primary cancer of the bone that occurs mainly in children and adolescents (Ritter and Bielack, 2010; Gianferante et al., 2017). It is derived from and defined by the presence of malignant mesenchymal cells that produce osteoid and/or immature bones (Arndt et al., 2012). Osteosarcomas generally attack the metaphyses of long tubular bones, and about half of them involve the distal femur or proximal tibia, which are located around the knee joints (Arndt and Crist, 1999). The 5-year survival rates and quality of life in patients with lung metastasis remain poor, despite developments and advances in treatments, including neoadjuvant chemotherapy 2.

Long non-coding RNAs (lncRNAs) (> 200 nucleotides) are a class of RNA transcripts that can regulate gene expression but cannot code protein (Bu et al., 2012; Kung et al., 2013). LncRNAs have been reported to play a role in a variety of biological pathways (Jiapaer et al., 2018; Song et al., 2019) and disease states (Kung et al., 2013), including tumors (Gutschner and Diederichs, 2012). Recently, the role of lncRNAs in osteosarcoma pathogenesis has attracted much attention and is being heavily investigated, particularly through high-throughput sequencing. Several of these lncRNAs are implicated in the downregulation of target gene expression (Yang et al., 2019), while others have been found to upregulate the expression of target genes (Li et al., 2013; Deng et al., 2019). An increasing number of studies indicate that lncRNAs play an important role in osteosarcoma development by affecting the following processes: cell proliferation, cellular invasion, migration, metastasis, stemness, fate, and EMT (Huang et al., 2018; Zhang et al., 2018; Shen et al., 2019). Moreover, several lncRNAs are being actively investigated as prognostic markers for this disease (Chen et al., 2018; Zhang et al., 2018).

A mechanism of interest, which has been reported on several times, involves lncRNAs working as competing endogenous RNAs (ceRNA), in which the lncRNA can act as a sponge for specific miRNAs and indirectly regulate the expression of tumor-related genes (Zhang et al., 2018; Deng et al., 2019; Shen et al., 2019). Tp53, a well-known and extensively studied gene in tumors, was first reported to exhibit intron 1 rearrangement (Masuda et al., 1987). TP53 has also been shown to affect the progression and development of OS through the regulation of miRNA by lncRNAs (Zhang et al., 2019; Zhang et al., 2019).

Herein, we discovered a new ceRNA axis, comprising LINC00588/miRNA-1972/TP53, that is involved in the development of OS, and we believe that this may deepen our understanding of OS etiology and contribute to an improvement in treatment choices.

## Materials and Methods

### Database

We typed “osteosarcoma” and “metastases” as keywords in the GEO Datasets public domain database. After the analysis of the data, we found that the lncRNA LINC00588 is highly expressed in the osteosarcoma bone primaries but has low expression in lung metastases.

### Cell Lines and Cell Culture

U2OS, HOS, MG-63, HFF-1, Saos-2, hBMSCs, and hFOB1.19 cell lines were purchased from the Chinese Academy of Sciences Cell Bank (Shanghai, China). They were cultured in Dulbecco's modified Eagle's medium (DMEM; Gibco) containing 10% fetal bovine serum (FBS) and supplemented with 10^5^ U/L penicillin and 100 mg/L streptomycin under 5% CO_2_ at 37°C in a humidified atmosphere.

### Cell Transfection

U2OS and HOS cells were plated at 1*10^5 cells/well in six-well plates containing serum-free culture medium with polybrene (6 μg/ml). At 40–50% confluence, cells were transduced with pLVX-LINC00588, pLVX-Vector, pLKO.1-LINC00588, pLKO.1-Vector, mimic-miRNA-1972, mimic-NC, inhibitor-miRNA-1972, and inhibitor NC, separately.

### RNA Extraction and Quantitative Reverse Transcription PCR Assay

TRIzol Reagent (Invitrogen, Carlsbad, USA) was used to extract total RNA according to the manufacturer's instructions. RNA was quantified and reverse transcribed into cDNA using the PrimeScipt RT Master Mix kit (Takara, Shanghai, China). RT‐PCR of the mature miRNAs was performed using the miRcute miRNA First‐Strand cDNA Synthesis Kit (Tiangen, Shanghai, China). According to the user guide of the SYBR^®^ Green Realtime PCR Master Mix (Takara, Shanghai, China), the qRT‐PCR amplification was done on an ABI7500 Fast system. Melting curve analysis was used to monitor the specificity of the PCR products. The LINC00588 primers were as follows: forward, 5′‐GTGATGGCAACCTTTCACCG‐3′; reverse, 5′‐ TCGCCTGAATTAGGCTCCTG‐3′. The GAPDH primers were as follows: forward, 5′‐CGGAGTCAACGGATTT GGTCG‐3′; reverse, 5′ ‐TCTCGCTCCTGGAAGATGGTG AT‐3′. The U6 primers were as follows: forward, CTCGCTTCGGCAGCACA The GAPDH primers were as follows: reverse, AACGCTTCACGAATTTGCGT. The E-cadherin primers were as follows: forward, GGACAGCAACATCAGCGAAC; reverse, GTTGAGATAAGCCTAGTCTTCCAGA. The ZEB1 primers were as follows: forward, GGAGAGGTGACTGGTTGTGG; reverse GCCACATCAGCAATAGCAGC. The Snail primers were as follows: forward, CTCAGCAGGGTGGTTACTGG; reverse, TGTCACCAGGACAAATGGGG. The Fbronectin primers were as follows: forward, AAAGGAGCCCAGGGTGTGA; reverse, GGAAAAGTCCTGAGGTGGGG. All of these primers were purchased from Sangon Biotech, Shanghai, China. All experiments were performed in triplicate. Changes in expression were calculated using the 2^−ΔΔCt^ method, and U6 was used as internal control for mRNA expression.

### Western Blot Assay

Total protein was extracted from cells using a total protein extraction kit according to the manufacturer's instructions (KeyGen, Rockville, MD, USA). The protein concentrations of the lysates were measured with the Protein BCA Assay Kit (Bio-Rad, Hercules, CA, USA). For the Western blotting assay, 20 µg of protein mixed with 5 × SDS loading buffer was loaded per lane, separated with 10% SDS-PAGE, and transferred to a nitrocellulose membrane. The membrane was blocked with 5% skimmed milk in TBST (TBS containing 0.05% Tween-20) for 1 h at room temperature. The membrane was then incubated at 4°C overnight with primary antibodies against TP53 (1:500, abcam), and GAPDH (1:1500, Santa Cruz Biotechnology, Inc.) followed by incubation with goat anti-mouse IgG (1:1000, Beijing Biosynthesis Biotechnology Co., Ltd.) or goat anti-rabbit antibody IgG (1:1000, Beijing Biosynthesis Biotechnology Co., Ltd.) for 1 h at room temperature. ECL Western blotting detection reagents (New England Biolabs, Ipswich, MA, USA) were used to visualize the target proteins, which were quantified with a Bio Image Intelligent Quantifier 1-D (Version 2.2.1, Nihon-BioImage Ltd., Japan).

### CCK-8 Assay

After transfection with pLVX-vector, pLVX-LINC00588, pLKO.1-vector, pLKO.1-LINC00588, equal numbers of HOS and U2OS cells were seeded in 96-well plates respectively, and at 0, 24, 148 48, and 72 h post-transfection, cell proliferation was measured with Cell Counting Kit-8 (CCK8, R&S Bio-150 Technology, Shanghai, China) according to the manufacturer's instructions. The OD values were measured at a wavelength of 450 nm with a microplate spectrophotometer (Multiskan MK3).

### Cell Colony Formation Assay

Cell proliferation ability was also detected by colony formation assay. Human osteosarcoma cells were seeded into six-well plates at a density of 1000 cells/ml, 2 ml/well. The cells were transfected with pLVX-vector, pLVX-LINC00588, pLKO-vector, and pLKO-LINC00588. The medium was replaced with fresh medium every 3 days. After incubation for 10 days, the cells were fixed with 70% ethanol and stained with 0.1% crystal violet for 30 min at 37°C. Images of the colonies were captured by Quantity One (Bio-Rad Laboratories, Inc., Hercules, CA, USA).

### Transwell Assay

The migration and invasion potential of osteosarcoma cells was evaluated by transwell assay. At 48 h after transfection, cells were seeded in the upper chamber of each transwell chamber (Corning Costar, Corning, NY, USA) and maintained in serum-free medium. For invasion assay, the insert was coated with Matrigel, while, for the migration assay, the insert had no Materigel coating; the lower chamber contained the medium supplemented with 10% FBS. After incubation for 24 h, the cells remaining on the upper membrane were gently removed with a cotton swab, and then the cells on both sides of the chamber were fixed with methanol. All the cells that stuck to the lower surface of the membrane were dyed with crystal violet solution and then imaged and counted.

### Analysis of Cell Cycle and Apoptosis *via* Flow Cytometry

For the cell cycle analysis, the transfected cells were harvested and stained with propidium iodide using a Cell Cycle Analysis kit (Biyuntian; Jiangsu, china), followed by assessment using flow cytometry. Using FlowJo software 7.6 (Tree Star, Inc., Ashland OR, USA), the percentage of cells in different phases was counted. An FITC Annexin V Apoptosis detection kit (Bd Biosciences, Franklin Lakes, NJ, USA) was used to analyze apoptosis. The transfected cells were harvested and re-suspended in binding buffer, and Annexin V-FITC and propidium iodide were used to stain the cells. Flow cytometry was performed according to the manufacturer's protocol.

### Xenograft Model

HOS cells and U2OS cells stably expressing pLVX-vector, pLVX-LINC00588, pLKO.1-vector, or pLKO.1-LINC00588 were subcutaneously injected into either side of the flank area of 8‐week‐old female athymic nude mice (1 × 10^7 per mouse, n = 5 mice per group). All animal experiments were approved by the ethics committee of Xinhua Hospital, Shanghai Jiao Tong University School of Medicine. Tumor volumes were measured (0.5 × length×width^2) in mice every 1 week by caliper. After 3 weeks, the nude mice were sacrificed, and the tumor tissues were excised and weighed.

### Wound Healing Assay

The cells were implanted uniformly into six‐well plates. When the cell density achieved 80~85% coverage of the well bottom, the culture solution was discarded. A loading tip (model: 1000μl) was then applied to draw a straight line going as much as possible through the area with the highest cell density. After cleansing with PBS solution two times, 3–5 ml serum‐free medium was again added. Finally, the cells were cultivated for 24 hr, after which cell migration was observed under the microscope.

### Luciferase Reporter Assay

To determine whether miRNA-1972 directly targets LINC00588 and TP53, we constructed wild plasmids (LINC00588‐wt and TP53‐wt) and mutant plasmids (LINC00588‐mt and TP53‐mt) expressing the site for miRNA-1972 to bind with LINC00588 and TP53 and then transfected them into U2OS and HOS cells, respectively. miRNA-1972 mimics or miRNA NC were also transfected by using Lipofectamine 2000 (Invitrogen, USA). Twenty‐four hours before transfection, the cells were seeded at 1.5 × 10^4^/well in 96‐well plates. The luciferase assay was performed with the Dual‐Luciferase Reporter Assay System (Promega) 48 hours after transfection.

### RNA Pull-Down Assay

Bio-labeled miR-1972 (Bio-miR-1972) and bio-labeled negative control (Bio-NC) were obtained from Sangon (Shanghai, China). The lysate samples of U2OS and H2OS cells were incubated with Bio-miR-1972 or Bio-NC. Then, the RNA-RNA complex was conjugated with streptavidin magnetic beads. After elution, the level of LINC00588 was measured by qRT-PCR.

### RNA Immunoprecipitation Assay

RNA immunoprecipitation (RIP) was applied to validate the direct binding between LINC00588 and miRNA-1972 using the EZMagna RIP kit (Millipore) according the manufacturer's protocol. The OS cells were transfected with LINC00588 for 48 h and then incubated with anti-MS2bs antibody and IgG (Millipore). Samples were incubated with Proteinase K, with shaking to digest the protein. qRT-PCR was used to detect the expression of miRNA-1972.

### Statistical Analysis

All data were presented as mean ± SD. All experiments were repeated at least three times. Comparison of two experimental groups was performed by the unpaired Student's t-test. All statistical analyses were conducted by the SPSS software version 17.0. P < 0.05 was considered to be statistically significant.

## Results

### Expression of the lncRNA LINC00588

**Figure 1:**
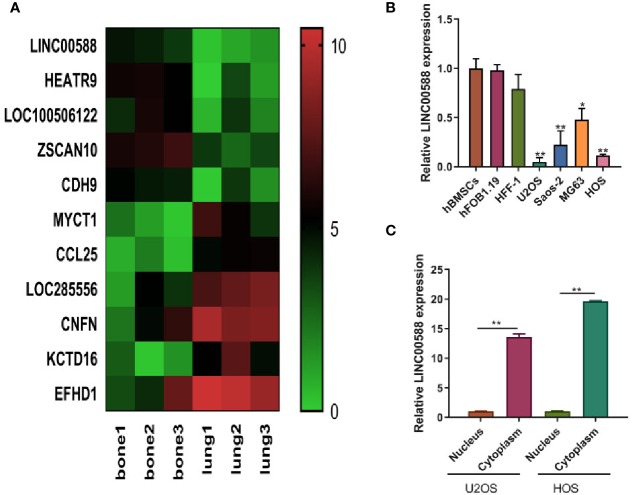
The expression of lncRNA LINC0058 (GEO ID: GSE85537). **(A)** A heat map representing the differential expression between the OS in bone and the metastatic tumor in lung. **(B)** The lncRNA LINC00588 has lower expression in the U2OS and HOS cell lines (* P < 0.05 ; **0.01 < p < 0.05 when compared to the hBMSC group). **(C)** The lncRNA LINC00588 is more highly expressed in the cytoplasm than in the nucleus in both the U2OS and HOS cell lines (**0.01 < p < 0.05).

Current knowledge in the field has detailed the involvement of lncRNAs in several cancer-related pathways, and the role of lnCRNAs in OS is being increasingly appreciated. Based on the data (GSE85537) from GEO Datasets, we observed that LINC00588 was expressed at a higher level in bone than at the metastatic site in lung (**Figure 1A**). Subsequently, we evaluated the expression of LINC00588 by qRT-PCR in a variety of cell lines and found that LINC00588 expression was lower in the HOS and U2OS cell lines (**Figure 1B**). To further study the subcellular localization of LINC00588 in HOS and U2OS, we separated the nuclear and cytoplasmic RNA fractions and found that the LINC00588 was mainly expressed in the cytoplasm in both cell lines (**Figure 1C**).

### Biological Function of the lncRNA LINC00588 in OS Cells

**Figure 2:**
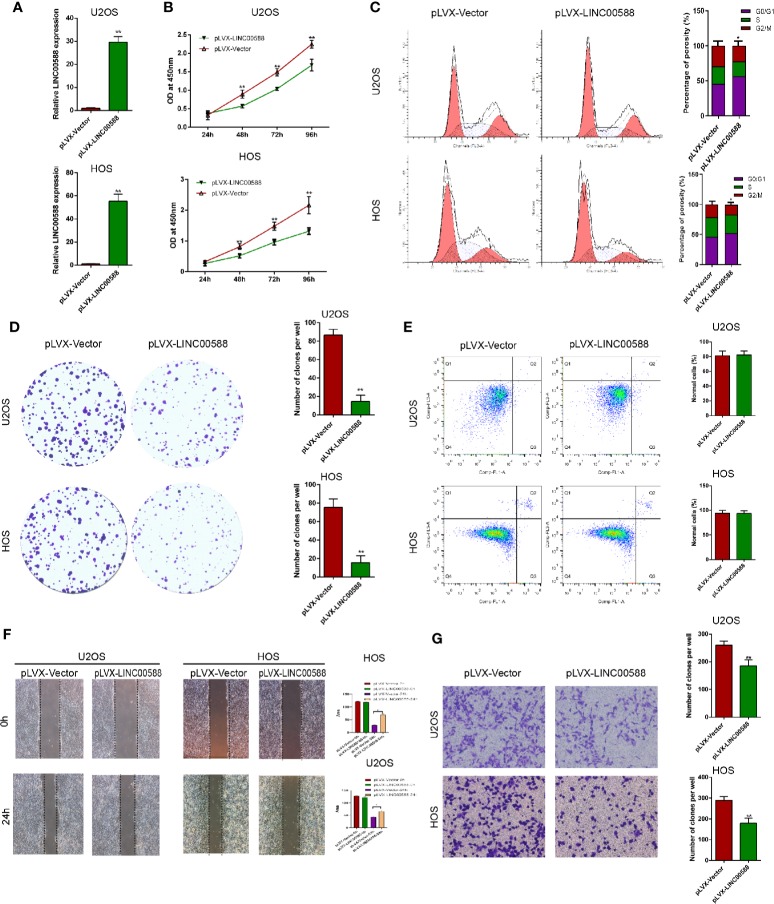
LINC00588 overexpression inhibits the proliferation and invasion of osteosarcoma. **(A)** Compared to when they were transfected with pLVX-VECTOR, the expression of lncRNA LINC00588 was significantly upregulated in both HOS and U2OS cells after being transfected with pLVX-LINC00588 (**0.01 < p < 0.05). **(B)** Cell viability was reduced following transfection with pLVX-LINC00588 in U2OS and HOS cells at different time points compared to under transfection with pLVX-VECTOR(**0.01 < p < 0.05 when compared to the 24 h group). **(C)** Cell populations of the U2OS and MG63 cells at different cell−cycle stages were analyzed *via* flow cytometry following transfection with pLVX-LINC00588 and pLVX-VECTOR, respectively (*P < 0.05). **(D)** Cell colony formation was weakened after transfection with pLVX-LINC00588 in both HOS and U2OS cells. **(E)** Both HOS and U2OS cells have no alternation after being transfected with pLVX-LINC00588. **(F, G)** Cell invasion and migration of HOS and U2OS after transfection with pLVX-LINC00588 were determined by transwell assay and wound healing assay, respectively (*P < 0.05, **0.01 < p < 0.05).

**Figure 3:**
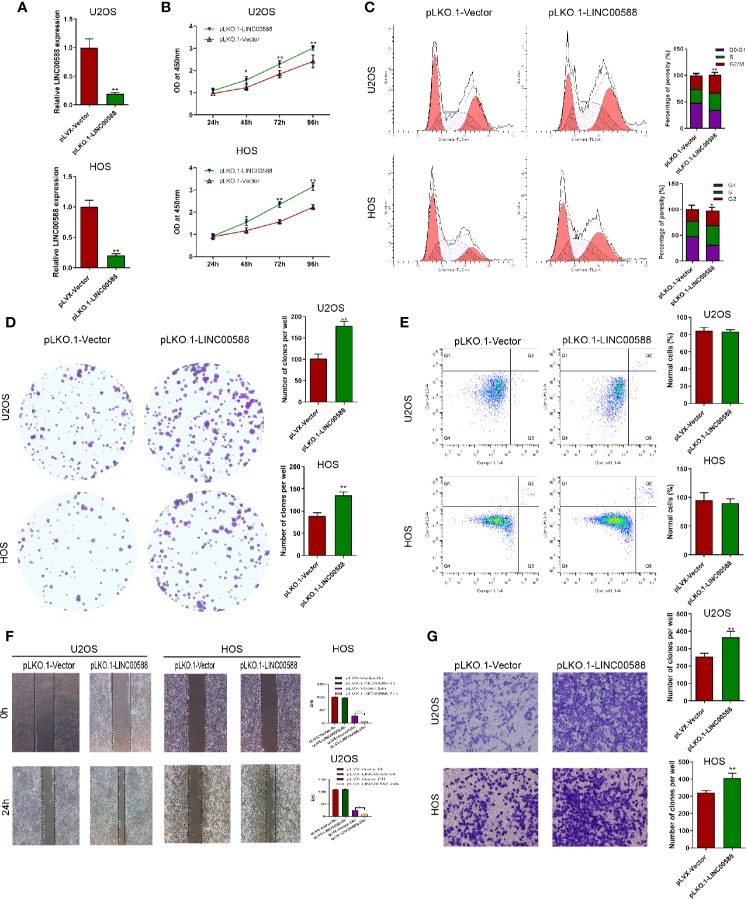
LINC00588 suppression promotes the proliferation and invasion of osteosarcoma. **(A)** Compared to under transfection with pLKO.1-VECTOR, the expression of the lncRNA LINC00588 was significantly downregulated in both HOS and U2OS cells after being transfected with pLKO.1-LINC00588 (**0.01 < p < 0.05). **(B)** Cell viability was enhanced following transfection with pLVX-LINC00588 in U2OS and HOS cells at different time points compared to under transfection with pLVX-VECTOR (**0.01 < p < 0.05). **(C)** Cell populations of the U2OS and MG63 cell lines at different cell−cycle stages were analyzed *via* flow cytometry following transfection with pLVX-LINC00588 and pLVX-VECTOR, respectively (*P < 0.05, **0.01 < P < 0.05). **(D)** Cell colony formation was enhanced after transfection with pLVX-LINC00588 in both HOS and U2OS cells (**0.01 < P < 0.05). **(E)** Both the HOS and U2OS cells show no alteration after being transfected with pLVX-LINC00588. **(F, G)** Cell invasion and migration of HOS and U2OS after being transfected with pLVX-LINC00588 were determined by Transwell assay and wound-healing assay, respectively (*P < 0.05, **0.01 < P < 0.05, ***P < 0.01).

To test the biological function of the lncRNA LINC00588 in the OS cells, we overexpressed LINC00588 by using either the lentiviral vector pLVX-LINC00588 or the pLVX-VECTOR, which was used as a control. The expression level of LINC00588 in the pLVX-LINC00588 group was increased ~30-fold when compared to the pLVX-VECTOR groups in both the HOS and U2OS cell lines (**Figure 2A**). The overexpression of LINC00588 resulted in an inhibition of viability, proliferation, migration, invasion, and arrested cell cycle in the G2 phase of OS (**Figures 2B–D, F, G**). Additionally, we knocked down LINC00588. The expression level of LINC00588 in the pLKO.1-LINC00588 group was about five times lower when compared to the pLKO.1-VECTOR group in both the HOS and U2OS cell lines (**Figure 3A**). The knock down of LINC00588 resulted in an enhancement of cell viability, proliferation, migration, invasion, and arrested cell cycle in the G2 phase of OS cells (**Figures 3B–D, F, G**). LINC00588 seems not to affect cell apoptosis (**Figures 2E** and **3E**).

### LINC00588 Regulates Endothelial Cell Function

**Figure 4:**
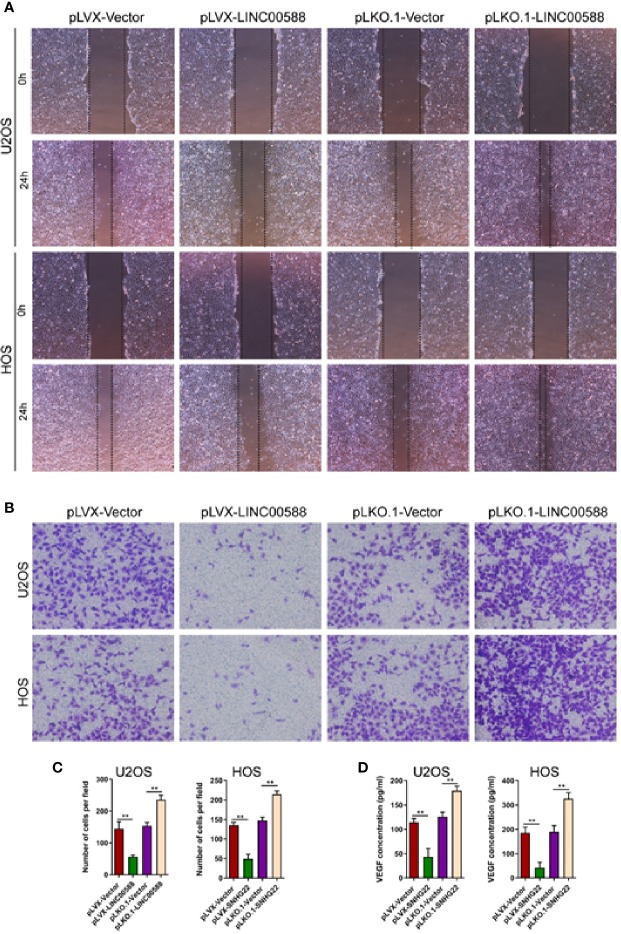
LINC00588 regulates endothelial cell function. **(A)** Scratch assay to detect the effect of U2OS and HOS cell culture supernatant on the migration of HUVEC. **(B)** Transwell assay to detect the effect of U2OS and HOS cell culture supernatant on the invasion of HUVEC. **(C)** Quantitative detection of the effect of U2OS and HOS cell culture supernatant on invasion of HUVEC cells. **(D)** ELISA assay to detect the expression of VEGF in the U2OS and HOS cell culture supernatant. (**0.01 < p < 0.05).

We performed a scratch assay using cell culture supernatants from U2OS and HOS cell culture supernatants and assessed their impact on the HUVEC cells. We showed that cell culture supernatants from U2OS and HOS cells that were overexpressing LINC00588 significantly reduced the migration and invasion of HUVECs cells (**Figures 4A–C**), while we obtained the opposite results after we knocked down LINC00588 (**Figures 4A–C**). We also performed ELISAs, which showed that an over-expression of LINC00588 could effectively inhibit the expression of vascular endothelial growth factor (VEGF) in U2OS and HOS cells, while its expression increased after we knocked down LINC00588 (**Figure 4D**).

### Effect of LINC00588 on EMT of OS Cells

**Figure 5:**
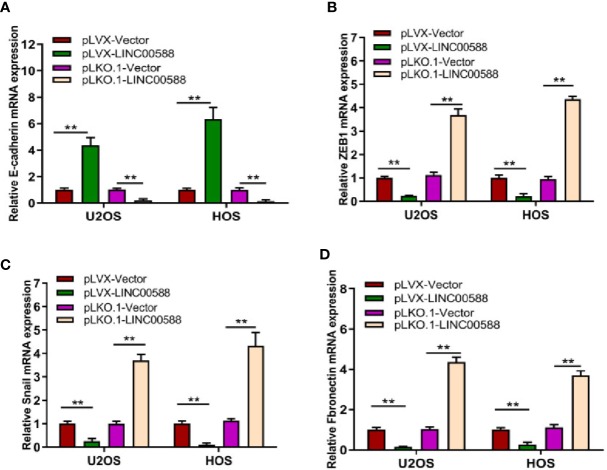
LINC00588 regulates the epithelial to mesenchymal transition (EMT) of OS cell lines. The relative expressions of EMT mRNA, E-cadherin **(A)**, ZEB1 **(B)**, Snail **(C)**, and Fibronectin **(D)** were detected by qPCR (**0.01 < p < 0.05).

Epithelial to mesenchymal transition (EMT), whereby epithelial cells are transformed into mesenchymal cells, is associated with tumor metastasis (Thiery et al., 2009; Kalluri and Weinberg, 2009). Given the differential expression of LINC00588 between the OS in bone and the metastatic tumor in lung, we attempted to test the effect of LINC00588 expression on the EMT of OS cells. As seen in **Figure 5**, the relative gene expression of EMT-related gene E-cadherin (A) was upregulated in the pLVX-LINC00588 group while being downregulated in the pLKO.1-LINC00588 group when compared to their respective control groups. The relative gene expression of EMT-related genes ZEB1(B), Snail(C), and Fibronectin(D) were significantly decreased in the pLVX-LINC00588 group while being correspondingly increased in the pLKO.1-LINC00588 group when compared to their respective NC groups.

### Relationship Between LINC00588, miRNA-1972, and TP53 Within Osteosarcoma Cells

**Figure 6:**
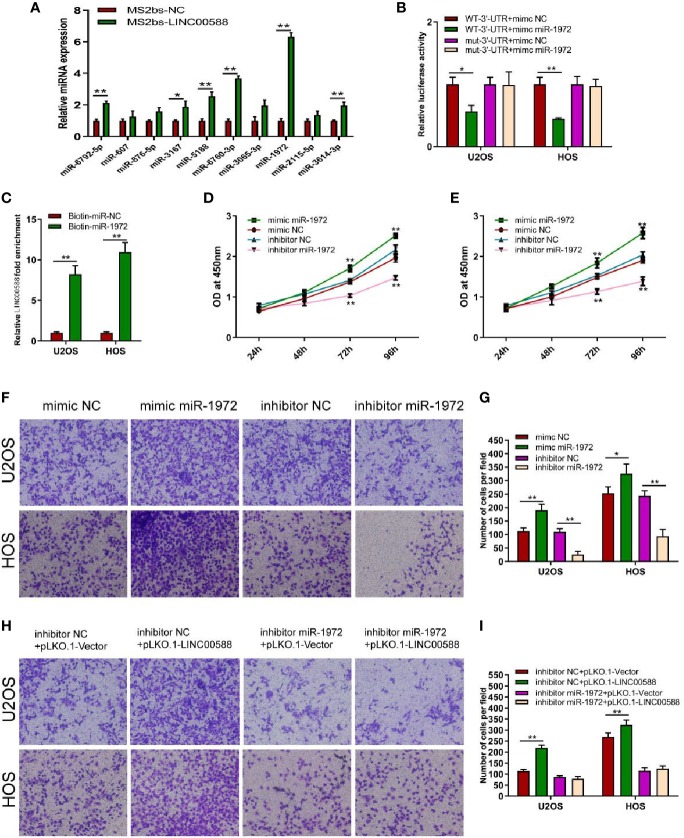
Regulation of miRNA-1972 by the lncRNA LINC00588 in OS cells. **(A)** miRNA-1972 was verified to combine with LINC00588 through RIP assay. (*P < 0.05, **0.01 < p < 0.05). **(B)** The connection between the lncRNA LINC00588 and miRNA-1972 was examined by luciferase reporter assay (*P < 0.05, **0.01 < p < 0.05). **(C)** RNA pull-down confirmed the interaction between LINC00588 and miRNA-1972 (*P < 0.05, **0.01 < p < 0.05). **(D, E)** The viability of OS cells was enhanced in the mimic miR-1972 group but inhibited in the inhibitor miR-1972 group compared to the respective NC group in both the HOS and U2OS cell lines. (**0.01 < p < 0.05). **(F–I)** The invasion of OS cells was enhanced in the mimic miR-1972 group and inhibitor+pLKO.1-LINC00588 group while being inhibited in the inhibitor miR-1972 compared to NC groups. The invasion of OS cells showed no difference between the inhibitor miR-1972+ pLKO.1-vector group and the inhibitor miR-1972+ pLKO.1-LINC00588 group (*P < 0.05, **0.01 < p < 0.05).

**Figure 7:**
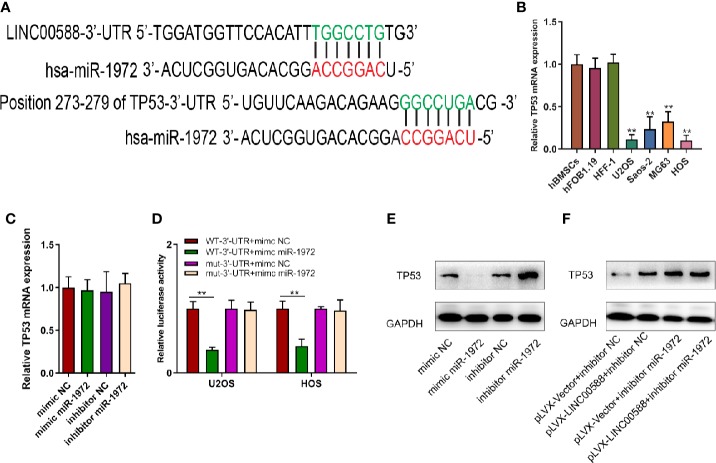
The correlation between miR‐1972 and TP53. **(A, D)** miR‐1972 targeted TP53 in certain sites, and the luciferase activities within osteosarcoma cell lines were compared between the WT group and the mutant group. **0.05 < p < 0.01 when compared with the WT-3'-UTR+mimic NC + WT-3'-UTR+mimic miR-1972 group. **(B)** TP53 has lower expression in the U2OS and HOS cell lines. **(C, E, F)** The expression of TP53 at the mRNA and protein levels was determined after alteration of LINC00588 expression or miR‐1972 expression. GAPDH, glyceraldehyde 3‐phosphate dehydrogenase; NC, negative control.

To determine the interaction between LINC00588, miRNA-1972, and TP53, we used both the miRDB and TargetScan databases to examine the presence of any potential complementary sequences between the three molecules (**Figure 7A**). To verify this prediction, RNA immunoprecipitation (RIP) assay was performed to verify the interaction between LINC00588 and miR-1972 (**Figure 6A**). Furthermore, dual-luciferase report and RNA pull-down assays were employed. The outcomes of dual-luciferase reporter analysis showed that the luciferase activity was significantly impaired in the WT + mimic miR-1972 group compared with the WT + mimic NC group, while there was no distinct change in the mut group (**Figure 6B**). As presented in **Figure 6C**, the level of LINC00588 was much more enriched in the Bio-miR-1972 group compared to in the Bio-NC group. These results convincingly supported that LINC00588 acts as a miR-1972 sponge in U2OS and HOS cell lines.

**Figure 8:**
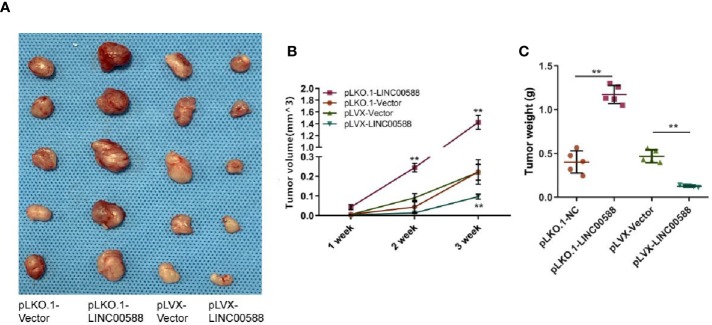
Overexpression of LINC00588 suppressed the growth of OS *in vivo*. **(A)** Representative images of tumors formed by nude mouse xenograft of pLKO.1-vector, pLKO.1-LINC00588, pLVX-Vector, and pLVX-LINC00588 OS cells (each group n = 5). **(B)** Summary of tumor volumes of mice, which were measured every 1 week. **(C)** Plot of tumor weights under tumor formation by nude mouse xenograft of pLKO.1-Vector, pLKO.1-LINC00588, pLVX-Vector and pLVX-LINC00588 OS cells (each group n = 5). (**0.01 < p < 0.05).

Next, we tried to verify the correlation between miRNA-1972 and TP53. It is not hard to see that TP53 had lower expression in both the HOS and U2OS cell lines (**Figure 7B**). We then wanted to evaluate whether either LINC00588 or miRNA-1972 could affect the expression of TP53. We therefore tested the expressions of TP53 at the mRNA and protein levels through qPCR and Western blot assays, respectively. The mRNA levels of TP53 remained unaltered (**Figure 7C**), while TP53 was upregulated after knockdown of miRNA-1972 at the protein level (**Figure 7E**). On the other hand, as **Figure 7F** shows, LINC00588 could neutralize the effect of miRNA-1972 to some extent. Furthermore, we verified this finding *in vivo*. LINC00588 knockdown significantly increased the tumor volume and weight in U20S cell lines compared with control groups, while the overexpression of LINC00588 could reduce the tumor volume and weight of U20S cell lines compared with control groups. (**Figures 8A–C**). These data revealed that LINC00588 was involved in tumorigenesis both *in vitro* and *in vivo*.

### Effect of LINC00588 Sponging the miRNA-1972 in OS Cells

Since we have demonstrated that LINC00588 could act as a sponge for miRNA-1972, we decided to test the effect of miRNA-1972 on OS cells. Therefore, we transfected the mimic miRNA-1972, mimic-NC, inhibitor miRNA-1972, and inhibitor NC into the HOS and U2OS cell lines. We performed a CCK-8 assay and transwell assay to show that overexpression of miRNA-1972 could enhance cell viability and invasion (**Figures 6D–G**). Subsequently, we performed a series of gain- and loss-of-function experiments. As shown by **Figures 6H, I**, the strengthening of invasion of OS cells caused by a downregulation of LINC00588 expression could be neutralized by utilizing the inhibitor of miRNA-1972.

## Discussion

Osteosarcoma is a multi-factorial disease and has been a hotspot of research for many years. Since lncRNAs were found to be involved in the process of tumorigenesis, the role of the lncRNAs in OS has been intensively studied. Originally, it was shown that a few lncRNAs are abnormally expressed in osteosarcoma (Li et al., 2013; Li et al., 2016). Subsequently, a large number of lncRNAs have been demonstrated to play an important role in the activities of osteosarcoma (Li et al., 2018; Huang et al., 2018; Deng et al., 2019). Furthermore, some lncRNAs have been demonstrated to be associated with the chemoresistance and overall survival of patients (Zhu et al., 2015; Chen et al., 2018; Chen et al., 2018). Therefore, we believe that further understanding the role of lncRNAs in the pathogenesis of osteosarcoma is of utmost importance.

The lung is the most common metastatic site in osteosarcoma, and therefore we have reason to believe that differential expression of lncRNAs between the OS and its metastatic tumor in lung may be associated with tumor progression. We found on the basis of GEO Datasets that, for osteosarcoma, the lncRNA ‘LINC00588' has low expression in bone but high expression its pulmonary metastatic tumor. Therefore, we believe that LINC00588 must play an important in the development of OS. Additionally, LINC00588 was expressed at a lower level in the HOS and U2OS cell line when compared to other OS and non-OS cell lines. We determined the role of LINC00588 by studying the effects of both overexpression and knockdown of LINC00588. LINC00588 could affect the viability, cell cycle, migration, and invasion but not the apoptosis of OS cells. Moreover, our data indicated that LINC00588 may regulate the endothelial cell function and EMT in the OS cell lines. However, the mechanisms of the above processes are still unclear.

LncRNAs can be grouped into five broad but mutually nonexclusive categories, which are “stand-alone lncRNAs,” “natural antisense transcripts,”“ Pseudogenes,” “long intronic ncRNAs,” and “Divergent transcripts, promoter-associated transcripts, and enhancer RNAs” (Kung et al., 2013). Recently, the action of lncRNA as a ceRNA that sponges one or more miRNAs has been studied extensively. Cao et al. indicated that lncRNA HOXA11-AS could work as a ceRNA by sponging miR-125a-5p and then regulate Rab3D expression, which is the basic component and major regulator of the vesicular transport signaling pathway, ultimately promoting the metastasis of OS (Cao et al., 2019). Similarly, Xu et al. found that lncRNA SNHG4 could promote tumor growth by sponging miR-224-3p (Xu et al., 2018). These results suggested that lncRNA LINC00588 may affect OS through sponging a specific miRNA.

Next, we predicted the target miRNA of the lncRNA using the miRDB database and verified it to be miRNA-1972 *via* luciferase and RIP assay. We also verified the ability of miRNA-1972 to affect cell viability and invasion. Cell viability was enhanced after overexpression of miRNA-1972, and viability was reduced after miRNA-1972 knockdown in both the HOS and the U2OS cell lines. Subsequently, we wanted to evaluate the influence of LINC00588/miRNA-1972 expression at the protein level and used the TargetScan database to predict that TP53 may work as a molecule for miRNA-1972. We verified this by performing a luciferase assay.

TP53 has become the most studied human gene since it was first recognized as a tumor suppressor in 1989 (Hafner et al., 2019). It was also found to be the most frequently mutated gene in tumorigenesis (Lawrence et al., 2014). Tp53 was first reported to hold intron 1 rearrangement by Masyda et al. (Masuda et al., 1987). A series of studies verified the presence of the mutation in TP53 in OS (Chen et al., 2014; Ribi et al., 2015; Sayles et al., 2019). In this study, we found that expression of TP53 was inhibited after overexpression of miRNA-1972, while TP53 expression was enhanced after overexpression of the lncRNA LINC00588 at the protein level.

In summary, our data show that the lncRNA LINC00588 is expressed in a different pattern in the cancer in bone and in the metastatic carcinoma in lung and that it may work as a ceRNA and finally affect the expression of TP53. Furthermore, our study highlights the potential of the LINC00588/miRNA-1972/TP53 axis as a therapeutic target in OS patients. However, more research needs to be performed to identify other pathways regulated by LINC00588 and to develop a further understanding of its role in both normal biology and disease physiology.

## Data Availability Statement

The datasets generated for this study are available on request to the corresponding authors.

## Ethics Statement

The animal study was reviewed and approved by the Research Ethics Committee of Xinhua Hospital.

## Author Contributions

All authors listed have made substantial, direct, and intellectual contribution to the work and approved it for publication. JSh, JSo, and F-CZ conceived and designed the study. F-CZ and H-TL performed the experiments. Y-HZ and H-TL wrote the paper. JSh and F-CZ received and edited the manuscript. All authors read and approved the manuscript.

## Conflict of Interest

The authors declare that the research was conducted in the absence of any commercial or financial relationships that could be construed as a potential conflict of interest.

## Abbreviations

hBMSC, human bone marrow-derived mesenchymal stem cell; ceRNA, competing endogenous RNA; lncRNA, long non-coding RNA; OS, osteosarcoma; TP53, tumor suppressor; EMT, epithelial to mesenchymal transition; NC, negative control.

## References

[B1] Arndt C. A. Crist W. M. (1999). Common musculoskeletal tumors of childhood and adolescence. N. Engl. J. Med. 341, 342–352. 10.1056/NEJM199907293410507 10423470

[B2] Arndt C. A. Rose P. S. Folpe A. L. Laack N. N. (2012). Common musculoskeletal tumors of childhood and adolescence. Mayo Clin. Proc. 87, 475–487. 10.1016/j.mayocp.2012.01.015 22560526 PMC3538469

[B3] Bu D. Yu K. Sun S. Xie X. Skogerbø G. Miao R (2012). NONCODE v3.0: integrative annotation of long noncoding RNAs. Nucleic Acids Res. 40, D210–D215. 10.1093/nar/gkr1175 22135294 PMC3245065

[B4] Cao K. (2019). The lncRNA HOXA11-AS regulates Rab3D expression by sponging miR-125a-5p promoting metastasis of osteosarcoma. Cancer Manag. Res. 11, 4505–4518. 10.2147/CMAR.S196025 31191012 PMC6529177

[B5] Chen X. Bahrami A. Pappo A. Easton J. Dalton J. Hedlund E. (2014). Recurrent somatic structural variations contribute to tumorigenesis in pediatric osteosarcoma. Cell Rep. 7, 104–112. 10.1016/j.celrep.2014.03.003 24703847 PMC4096827

[B6] Chen Z. X. Chen C. P. Zhang N. Wang T. X. (2018). Low-expression of lncRNA FER1L4 might be a prognostic marker in osteosarcoma. Eur. Rev. Med. Pharmacol. Sci. 22, 2310–2314. 10.26355/eurrev_201804_14820 29762833

[B7] Chen D. (2018). Abnormally expressed long non-coding RNAs in prognosis of Osteosarcoma: A systematic review and meta-analysis. J. Bone Oncol. 13, 76–90. 10.1016/j.jbo.2018.09.005 30591861 PMC6303364

[B8] Deng R. Zhang J. Chen J. (2019). lncRNA SNHG1 negatively regulates miRNA1013p to enhance the expression of ROCK1 and promote cell proliferation, migration and invasion in osteosarcoma. Int. J. Mol. Med. 43, 1157–1166. 10.3892/ijmm.2018.4039 30592267 PMC6365036

[B9] Gianferante D. M. Mirabello L. Savage S. A. (2017). Germline and somatic genetics of osteosarcoma - connecting aetiology, biology and therapy. Nat. Rev. Endocrinol. 13, 480–491. 10.1038/nrendo.2017.16 28338660

[B10] Gutschner T. Diederichs S. (2012). The hallmarks of cancer: a long non-coding RNA point of view. RNA Biol. 9, 703–719. 10.4161/rna.20481 22664915 PMC3495743

[B11] Hafner A. Bulyk M. L. Jambhekar A. Lahav G. (2019). The multiple mechanisms that regulate p53 activity and cell fate. Nat. Rev. Mol. Cell Biol. 20, 199–210. 10.1038/s41580-019-0110-x 30824861

[B12] Huang Q. Yang J. He X. Shi S. Xing S. (2018). LncRNA BDNF-AS is associated with the malignant status and regulates cell proliferation and apoptosis in osteosarcoma. Biosci. Rep. 38 (6) 10.1042/BSR20181498 PMC6240721 30352834

[B13] Jiapaer Z. (2018). LincU Preserves Naive Pluripotency by Restricting ERK Activity in Embryonic Stem Cells. Stem Cell Rep. 11, 395–409. 10.1016/j.stemcr.2018.06.010 PMC6092693 30017820

[B14] Kalluri R. Weinberg R. A. (2009). The basics of epithelial-mesenchymal transition. J. Clin. Invest. 119, 1420–1428. 10.1172/JCI39104 19487818 PMC2689101

[B15] Kung J. T. Colognori D. Lee J. T. (2013). Long noncoding RNAs: past, present, and future. Genetics 193, 651–669. 10.1534/genetics.112.146704 23463798 PMC3583990

[B16] Lawrence M. S. (2014). Discovery and saturation analysis of cancer genes across 21 tumour types. Nature 505, 495–501. 10.1038/nature12912 24390350 PMC4048962

[B17] Li J. Liu L. H. Chen Y Jiang X. W. Ouyang Y. R. (2013). Microarray expression profile of long noncoding RNAs in human osteosarcoma. Biochem. Biophys. Res. Commun. 433, 200–206. 10.1016/j.bbrc.2013.02.083 23466354

[B18] Li Z. Yu X. Shen J. (2016). Long non-coding RNAs: emerging players in osteosarcoma. Tumour Biol. 37, 2811–2816. 10.1007/s13277-015-4749-4 26718212

[B19] Li Z. Wang Y. Hu R. Xu R. Xu W. (2018). LncRNA B4GALT1-AS1 recruits HuR to promote osteosarcoma cells stemness and migration via enhancing YAP transcriptional activity. Cell Prolif. 51, e12504. 10.1111/cpr.12504 30182452 PMC6528912

[B20] Masuda H. Miller C. Koeffler H. P. Battifora H. Cline M. J. (1987). Rearrangement of the p53 gene in human osteogenic sarcomas. Proc. Natl. Acad. Sci. U. S. A. 84, 7716–7719. 10.1073/pnas.84.21.7716 2823272 PMC299371

[B21] Ribi S. Baumhoer D. Lee K. Teo A S. Madan B. Zhang k. (2015). TP53 intron 1 hotspot rearrangements are specific to sporadic osteosarcoma and can cause Li-Fraumeni syndrome. Oncotarget 6, 7727–7740. 10.18632/oncotarget.3115 25762628 PMC4480712

[B22] Ritter J. Bielack S. S. (2010). Osteosarcoma. Ann. Oncol. 21 (Suppl 7), vii320–vii325. 10.1093/annonc/mdq276 20943636

[B23] Sayles L. C. Bresse M R. Koehne A L. Leung S G. Lee A G. Liu H Y. (2019). Genome-Informed Targeted Therapy for Osteosarcoma. Cancer Discov. 9, 46–63. 10.1158/2159-8290.CD-17-1152 30266815 PMC7134333

[B24] Shen B. (2019). LncRNA MEG3 negatively modified osteosarcoma development through regulation of miR-361-5p and FoxM1. J. Cell Physiol. 234, 13464–13480. 10.1002/jcp.28026 30624782 PMC13483347

[B25] Song A. Feng R. Gao J. Yang C. (2019). Long noncoding RNA Alu-mediated p21 transcriptional regulator promotes proliferation, migration, and pipe-formation of human microvascular endothelial cells by sponging miR-126. J. Cell Biochem. 120 (12). 10.1002/jcb.29291 31310378

[B26] Thiery J. P. Acloque H. Huang R. Y. Nieto M. A. (2009). Epithelial-mesenchymal transitions in development and disease. Cell 139, 871–890. 10.1016/j.cell.2009.11.007 19945376

[B27] Xu R. Feng F. Yu X. Liu Z. Lao L. (2018). LncRNA SNHG4 promotes tumour growth by sponging miR-224-3p and predicts poor survival and recurrence in human osteosarcoma. Cell Prolif. 51, e12515. 10.1111/cpr.12515 30152090 PMC6528889

[B28] Yang Q. Yu H. Yin Q. Hu X. Zhang C. (2019). lncRNA-NEF is downregulated in osteosarcoma and inhibits cancer cell migration and invasion by downregulating miRNA-21. Oncol. Lett. 17, 5403–5408. 10.3892/ol.2019.10276 31186758 PMC6507433

[B29] Zhang R. M. Tang T. Yu H. M. Yao X. D. (2018). LncRNA DLX6-AS1/miR-129-5p/DLK1 axis aggravates stemness of osteosarcoma through Wnt signaling. Biochem. Biophys. Res. Commun. 507, 260–266. 10.1016/j.bbrc.2018.11.019 30442366

[B30] Zhang Y. Meng W. Cui H. (2018). LncRNA CBR3-AS1 predicts unfavorable prognosis and promotes tumorigenesis in osteosarcoma. BioMed. Pharmacother. 102, 169–174. 10.1016/j.biopha.2018.02.081 29554595

[B31] Zhang G. D. Gai P. Z. Liao G. Y. Li Y. (2019). LncRNA SNHG7 participates in osteosarcoma progression by down-regulating p53 via binding to DNMT1. Eur. Rev. Med. Pharmacol. Sci. 23, 3602–3610. 10.26355/eurrev_201905_17782 31114984

[B32] Zhang Z. F. Xu H. H. Hu W. H. Hu T. Y. Wang X. B. (2019). LINC01116 promotes proliferation, invasion and migration of osteosarcoma cells by silencing p53 and EZH2. Eur. Rev. Med. Pharmacol. Sci. 23, 6813–6823. 10.26355/eurrev_201908_18720 31486480

[B33] Zhu K. P. Zhang C. L. Shen G. Q. Zhu Z. S. (2015). Long noncoding RNA expression profiles of the doxorubicin-resistant human osteosarcoma cell line MG63/DXR and its parental cell line MG63 as ascertained by microarray analysis. Int. J. Clin. Exp. Pathol. 8, 8754–8773. 26464619 PMC4583851

